# A comprehensive epigenetic network can influence the occurrence of thyroid-associated ophthalmopathy by affecting immune and inflammatory response

**DOI:** 10.1038/s41598-024-64415-8

**Published:** 2024-06-12

**Authors:** Zhuo Zhang, Hongshi Wu, Xun Gong, Yuerong Yan, Xiaohui Li, Rongxue Yang, Muchao Wu, Mingtong Xu

**Affiliations:** Department of Endocrinology, Sun Yat-sen Memorial Hospital, Sun Yat-sen University, Guangzhou, China

**Keywords:** Thyroid-associated ophthalmopathy, DNA methylation, Non-coding RNA, RNA methylation, Immune and inflammatory response, Genetics, Endocrinology

## Abstract

The primary objective of this study is to understand the regulatory role of epigenetics in thyroid-associated ophthalmopathy (TAO) using multi-omics sequencing data. We utilized tRFs sequencing data, DNA methylation sequencing data, and lncRNA/circRNA/mRNA sequencing data, as well as several RNA methylation target prediction websites, to analyze the regulatory effect of DNA methylation, non-coding RNA, and RNA methylation on TAO-associated genes. Through differential expression analysis, we identified 1019 differentially expressed genes, 985 differentially methylated genes, and 2601 non-coding RNA. Functional analysis showed that differentially expressed genes were mostly associated with the PI3K signaling pathway and the IL17 signaling pathway. Genes regulated by DNA epigenetic regulatory networks were mainly related to the Cytokine-cytokine receptor interaction pathway, whereas genes regulated by RNA epigenetic regulatory networks were primarily related to the T cell receptor signaling pathway. Finally, our integrated regulatory network analysis revealed that epigenetics mainly impacts the occurrence of TAO through its effects on key pathways such as cell killing, cytokine production, and immune response. In summary, this study is the first to reveal a new mechanism underlying the development of TAO and provides new directions for future TAO research.

## Introduction

Epigenetics is a fascinating field of biology that focuses on the intricate mechanisms that regulate gene expression in relation to the genes themselves. There are many different types of epigenetic modifications, and DNA methylation is a key concept in this area. This refers to the addition of a methyl group to cytosine in DNA, which can affect gene expression in important ways. DNA methylation is involved in a range of biological processes, including the regulation of embryonic development, cell differentiation, and the synthesis of secondary metabolism. However, abnormal changes in DNA methylation have been linked to the development of various diseases such as tumors and Mediterranean anemia^1^. Non-coding RNAs are another important area of epigenetics, and they include various types of RNA which do not produce proteins. Non-coding RNAs, such as long non-coding RNAs and microRNAs, play a critical role in many biological processes, including the regulation of gene expression, RNA modification, and protein translation. They have been linked to the development of many diseases, including various cancers, cardiovascular diseases, and neurodegenerative diseases^2^. For instance, certain miRNAs have been associated with liver cancer, breast cancer^3^, and ovarian cancer^4^, while lncRNAs have been shown to be linked to cardiovascular diseases^5^, diabetes^2^, and breast cancer^6^. RNA methylation is another important area of epigenetic research, and it involves chemical modifications to RNA molecules that can interfere with their interactions with other molecules. One key focus of RNA methylation research is m6A, which regulates gene expression and plays a role in subsequent biological processes such as mRNA stability, transcription, and translation^7,8^. Abnormal regulation of RNA methylation has been associated with the development of tumors and age-related neurological diseases such as Alzheimer's disease^9^, Parkinson's disease^10^, and depression. Overall, epigenetics is a fascinating area of biology that is helping to illuminate the intricate ways that our genes are regulated and controlled.

Thyroid-associated ophthalmopathy (TAO), which is also known as Graves’ ophthalmopathy (GO) or thyroid eye disease (TED), is an autoimmune disease that is commonly seen in patients with hyperthyroidism and also occurs occasionally in euthyroid or hypothyroid patients, and patients often experience eye and surrounding tissue inflammation and edema. Its symptoms include mild eye irritation, severe disfigure and even permanent blindness. TAO is the result of a combination of genetic and environmental factors^11–13^. Although growing evidence suggests that the immune response is an important factor involved in TAO, the pathologic mechanism of TAO has not been elucidated in detail, and the main therapeutic strategies for TAO, such as corticosteroids, orbital irradiation, and surgical decompression, still have difficulties to achieve desired outcome for the treatment of TAO. Understanding the pathological mechanism of TAO will lead to the discovery of targeted molecular approaches for TAO and provide strong evidence for the future treatment of TAO^14–16^. Up to date, more and more studies show that epigenetics plays a crucial role in regulating thyroid eye disease, particularly microRNAs (miRNAs) and long non-coding RNAs (lncRNAs), these ncRNAs have the potential to be diagnostic markers for GO, which also play a key role in the regulation of inflammation, regulation of T cell functions, regulation of glycosaminoglycan aggregation and fibrosis, glucocorticoid sensitivity, lipid accumulation and adipogenesis, oxidative stress and angiogenesis, and proliferation^17^. According to one study, DNA methylation plays a role in regulating inflammatory receptors and basal metabolic rate in thyroid eye disease^18^. Furthermore, the decreased expression of histone deacetylase HDAC2 in eye tissue of patients with thyroid eye disease leads to the increased proliferation of T cells and worsening of inflammation^19^. Another research study has indicated that miR-133a, through the RhoA/ROCK signaling pathway, can influence cell contraction and proliferation, thereby affecting the occurrence and development of thyroid eye disease^20^.

Although there has been significant research on single omics and TAO, the disease is the result of the complex interaction of multiple omics. However, there is currently no research that comprehensively evaluates the impact of epigenetics on TAO. Therefore, our objective is to integrate sequencing data from multiple omics, including DNA methylation sequencing, RNA-seq, and tRFs sequencing, to examine the individual effects of different omics on TAO. In addition, we aim to comprehensively evaluate the overall regulatory effects of epigenetics on TAO by integrating different omics data. Finally, we plan to identify key genes that play a critical role in the regulatory mechanisms of epigenetics. The present work may promote the understanding of the pathological mechanism of TAO and serve as therapeutic targets or promise diagnostic biomarkers for TAO in the future studies.

## Material method

### Data usage

We conducted a thorough search for relevant datasets on the Gene Expression Omnibus (GEO) website. After careful evaluation, we identified three datasets that were pertinent to this study: GSE175399, which presents DNA methylation sequencing data which detects methylated cytosines by treating DNA with bisulfite, converting unmethylated cytosines to uracil, followed by sequencing to identify methylation patterns. RNA-seq, or RNA sequencing, is a powerful technique that employs next-generation sequencing (NGS) to capture and quantify the RNA present in a biological sample at a given moment. It enables the identification of gene expression levels, discovery of novel transcripts, alternative splicing events, and post-transcriptional modifications. By converting RNA into cDNA, which is then sequenced, RNA-seq provides a comprehensive view of the transcriptome, revealing insights into the functional complexity of genes and their regulatory networks. Based on the GEO database, we retrieved two sequencing datasets related to TAO.GSE186480 provides information on tRF (tRNA-derived fragments) expression; and GSE185952 features a comprehensive sequencing analysis of mRNA, lncRNA, and circRNA expression. Notably, the GSE185952 dataset comprises a 3 vs 3 expression profile data for mRNA, lncRNA, and circRNA, while GSE174399 illustrates a 4 vs 4 DNA methylation sequencing dataset. Finally, GSE186480 is a 3 vs 3 tRF expression chip.

### Difference analysis

We utilized the widely accepted standard pipeline of the CHAMP package^21^ to carefully analyze the DNA methylation sequencing data. This process included comprehensive steps for data filtering, normalization, and differential methylation analysis. As for the screening criteria for identifying differentially methylated regions, we employed the stringent and widely accepted thresholds of | logFC |> 0.2 and P < 0.05. Additionally, we carefully performed differential expression analysis using the limma algorithm^22^ on both RNA-seq and tRF sequencing data. Throughout this analysis, we implemented rigorous screening criteria of Log2 foldchange with an absolute value > 1 and P < 0.05.

### Epigenetic network construction

- DNA methylation regulatory network: First,we identify differentially methylated genes from the methylation data and differentially expressed genes from the expression data. Then find the intersection of genes that are highly expressed with low methylation and genes that are lowly expressed with high methylation to obtain genes regulated by methylation.
- Non-coding regulatory network: The Starbase database is a comprehensive resource designed for RNA interactions, offering detailed information on interactions between miRNA and a variety of RNA molecules (including lncRNA, circRNA, etc.). This database serves as the foundation for constructing a ceRNA (competitive endogenous RNA) network.Starbase database was used to predicted potential target genes, and then performed a cross-analysis with the differentially expressed mRNAs.
- RNA methylation regulatory network: RMBase (RNA Modification Base) is a comprehensive database designed to decipher the landscape of RNA modifications detected through high-throughput sequencing data. RM2Target is an extensive database that specifically examines the regulatory connections between RNA modification writers, erasers, readers, and their target genes. This database contains 1,619,653 associations between writers and genes, encompassing 63 writers and 9 types of RNA modifications in both humans and mice.We utilized two databases, RMBase^23^ and RM2Target^24^, to predict RNA methylation target genes for different types of RNA methylation, including M1A, PseudoU, m5u, m6Am, A-to-I, m7g, m5C, 2'-O-Methylation, and m6A. Through cross-analysis, our focus is on identifying the overlap between target genes and differentially expressed genes for each type of RNA methylation.

### Comprehensive epigenetic network construction

Using the DNA methylation, miRNA, and RNA methylation regulatory networks as a foundation, an integrated epigenetic regulatory network was created. The regulatory network is constructed using the cytoscape software3.10.2(https://cytoscape.org/). STRING (Search Tool for the Retrieval of Interacting Genes/Proteins) database is a comprehensive resource for analyzing protein–protein interaction (PPI) networks. The database is designed to collect, evaluate, and integrate all publicly available sources of protein–protein interaction information, supplemented by computational predictions to fill in missing data. STRING aims to provide a complete and objective global network that includes both direct (physical) and indirect (functional) interaction data. To further expand the network, protein–protein interactions between regulated genes were analyzed utilizing the STRING database (string-db.org)^25^. From this analysis, a protein–protein interaction network was constructed. Core modules within this regulatory network were identified through use of the MCODE algorithm.

### Functional analysis

Gene Ontology (GO) and the Kyoto Encyclopedia of Genes and Genomes (KEGG) database are used to annotate the functions of genes. To gain a deeper understanding of gene function within regulatory networks, the genes derived from these networks were subject to analysis using clusterprofiler^26^. This approach allowed for a thorough investigation into the functional properties of each gene, providing insight into their potential roles within a given system or network.

## Result

### TAO single omics feature analysis

**Figure 1:**
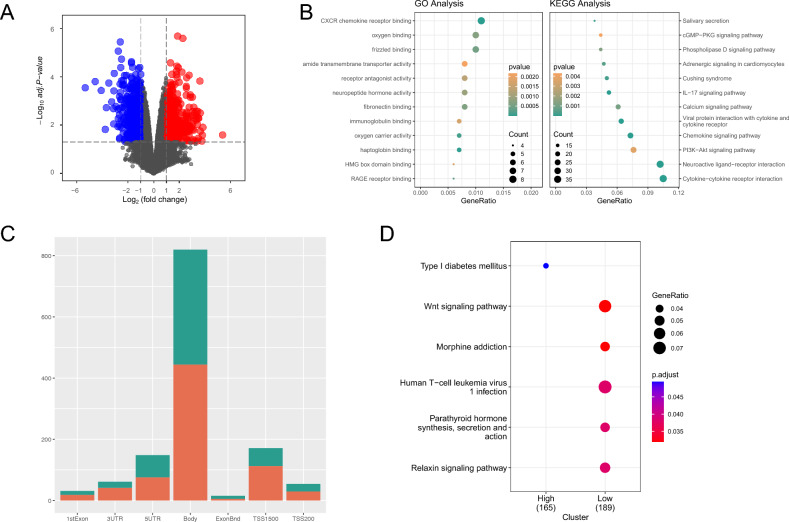
TAO omics analysis. (**A**) Volcano plot of mRNA expression analysis, where blue represents the downregulated genes, red represents the upregulated genes, and black represents the genes with no differential expression. (**B**) Bubble plot of differential gene enrichment analysis. The left side shows the GO analysis results, and the right side shows the KEGG pathway analysis results. The size of the bubble represents the number of differential genes involved in the pathway, and the color indicates the significance of these genes. (**C**) Statistics of differential methylation site locations, with red representing hypermethylated sites and green representing hypomethylated sites. (**D**) Enrichment analysis of differential high and low methylation sites, where high and low represent hypermethylation and hypomethylation, respectively. The size of the bubble indicates the number of differential genes involved in the pathway, and the color represents the significance of these genes.

Regarding mRNA expression, the RNA-seq data was analyzed for differential expression and it was found that there were 447 upregulated genes and 572 downregulated genes (Fig. 1A). The functional enrichment analysis of these genes showed that they play a role in CXCR chemokine receptor binding, oxygen binding, and other related functions. Additionally, KEGG pathway analysis revealed that these genes mainly play a role in signaling pathways such as cytokine-cytokine receptor interaction, PI3K-Akt signaling pathway, and IL-17 signaling pathway (Fig. 1B). For DNA methylation sequencing, a differential expression analysis was performed, which identified 512 hypermethylated sites and 473 hypomethylated sites. The site location analysis showed that most of the methylation (Supplementary Fig. 1) sites were located in the gene body, followed by TSS1500 and 5UTR (Fig. 1C). Enrichment analysis of hypermethylated and hypomethylated genes revealed that hypermethylated genes were mainly associated with type 1 diabetes related to endocrine function, whereas hypomethylated genes were related to multiple pathways, including the Wnt pathway and PI3K signaling pathway (Fig. 1D). Finally, the expression of circRNA and lncRNA was analyzed using GSE185952 and 1478 differentially expressed circRNAs and 1120 differentially expressed lncRNAs were identified. Additionally, GSE186480 was used for tRF expression analysis, which identified 50 tRFs, with three showing differential expression in TAO.

### DNA epigenetic network construction

**Figure 2:**
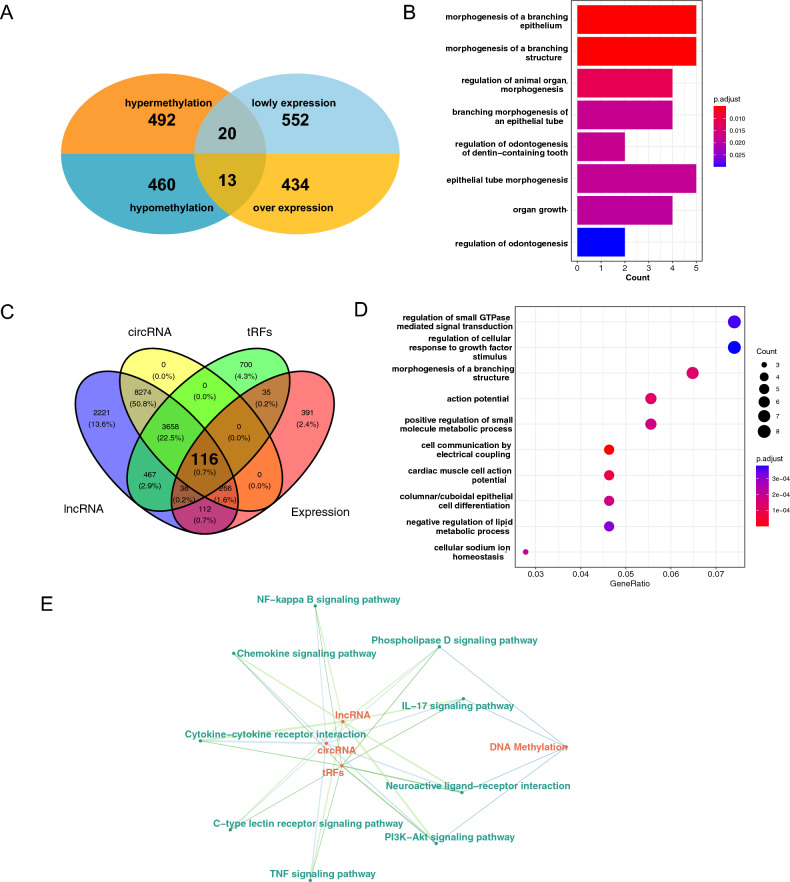
Construction of DNA epigenetic regulation network. (**A**) DNA methylation-regulated genes were analyzed to identify genes with negative regulation by DNA methylation. The high and low methylation-regulated genes with low expression and high expression were observed. (**B**) Bar chart of DNA methylation enrichment analysis, where the redder color indicates the more significant pathway. (**C**) Venn diagram of non-coding RNA-regulated genes, where the three circles (lncRNA, circRNA, tRFs) represent the genes regulated by each type of non-coding RNA, and Expression represents differentially expressed mRNA. The center region of the diagram represents the genes regulated by non-coding RNA. (**D**) Bubble plot of genes regulated by non-coding RNA enrichment analysis, where the size of the bubble indicates the number of differential genes involved in the pathway and the color represents the significance of these genes. (**E**) DNA epigenetic regulation network based on non-coding RNA and DNA methylation, where red represents different regulatory modes, and green labels represent the pathway of regulatory gene enrichment.

After performing cross-analysis, we discovered 13 hypomethylated-upregulated genes and 20 hypermethylated-downregulated genes (Fig. 2A). The enrichment analysis conducted for methylation-regulated genes highlighted that these 33 genes are primarily associated with branch epithelial morphogenesis and organ growth (Fig. 2B). Moving to our non-coding RNA target gene prediction, we initially utilized the Starbase database to forecast the lncRNA and circRNA target genes, while the tRFs database was employed to anticipate differentially expressed tRFs' target genes. We then carried out cross-analysis of the target genes with differentially expressed genes. This analysis enabled us to identify 116 genes regulated by non-coding RNA, with lncRNA regulating 532, circRNA regulating 382, and tRFs regulating 189 differentially expressed genes (Fig. 2C). These 116 genes show enrichment in pathways primarily associated with regulating the response of cells to growth factors (Fig. 2D). By combining the genes regulated at both levels, we were able to construct a DNA epigenetic regulatory network. We identified a total of 567 genes regulated by DNA epigenetics through this analysis. We also conducted functional enrichment analysis on these 567 genes to understand their major functions. Our analysis revealed that these genes mainly regulate the IL-17 signaling pathway (Table 1). Further evaluation of the regulatory function of DNA epigenetics revealed that DNA methylation is only involved in the regulation of four pathways, while Non-Coding RNA regulates almost all pathways. Notably, lncRNA regulates 27 genes in the Cytokine-cytokine receptor interaction pathway (Fig. 2E).

**Table 1:** The KEGG pathway analysis results.

ID	Description	Gene ratio	P value
hsa04657	IL-17 signaling pathway	16/258	2.9942E−08
hsa04061	Viral protein interaction with cytokine and cytokine receptor	16/258	7.4116E−08
hsa04060	Cytokine-cytokine receptor interaction	28/258	1.4955E−07
hsa05144	Malaria	10/258	2.7264E−06
hsa04062	Chemokine signaling pathway	18/258	3.2588E−05
hsa04668	TNF signaling pathway	13/258	4.6683E−05
hsa04080	Neuroactive ligand-receptor interaction	26/258	6.7938E−05
hsa04064	NF-kappa B signaling pathway	11/258	0.00040783
hsa05143	African trypanosomiasis	6/258	0.00094143
hsa04934	Cushing syndrome	13/258	0.00117833
hsa05120	Epithelial cell signaling in *Helicobacter pylori* infection	8/258	0.00151291
hsa04625	C-type lectin receptor signaling pathway	10/258	0.00155813
hsa04151	PI3K-Akt signaling pathway	22/258	0.00174567
hsa01523	Antifolate resistance	5/258	0.00222677
hsa04072	Phospholipase D signaling pathway	12/258	0.00240499
hsa05323	Rheumatoid arthritis	9/258	0.00253479
hsa04261	Adrenergic signaling in cardiomyocytes	12/258	0.00268984
hsa05412	Arrhythmogenic right ventricular cardiomyopathy	8/258	0.00279183

### RNA epigenetic network construction

**Figure 3:**
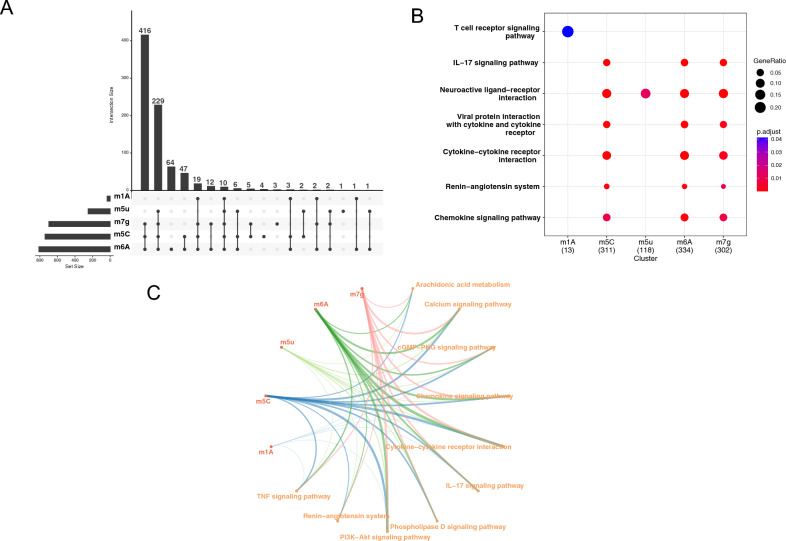
Construction of RNA epigenetic regulation network. (**A**) Upset plot of multiple RNA methylation-regulated genes, where the upper part of the plot represents the specific genes regulated by different RNA methylation. The left side represents the total number of genes regulated by RNA methylation. (**B**) Enrichment analysis of different RNA methylation. (**C**) RNA epigenetic regulation network, where red represents different RNA methylation patterns, and orange represents the pathway of enrichment analysis of RNA methylation-regulated genes.

We explored the different types of RNA methylation, including m6a, m7g, m1a, m5c, and m5u, by utilizing RMBase and R M2TARGET databases to predict target genes for each type of modification. We conducted a cross-analysis of these target genes with differentially expressed genes to identify differential genes in TAO. Our analysis revealed that m6A regulates a significantly higher number of genes compared to other RNA modifications, with a total of 812 genes, while m1A regulates a minimum of 35 genes (Fig. 3A). Among the other four types of RNA modifications, 229 genes were modified. Interestingly, we found a set of ten genes that were regulated by all five types of RNA modifications (Fig. 3B). Enrichment analysis of genes regulated by each modification showed that m1A mainly regulated the T cell receptor signaling pathway. In contrast, the remaining genes were found to be involved primarily in pathways similar to Neuroactive ligand-receptor interaction. By integrating the findings, we identified a total of 827 genes regulated by RNA modifications. Enrichment analysis of these genes unveiled that RNA modifications regulated 24 pathways, including the IL-17 signaling pathway (Table 2). Further analysis revealed that m6A modification plays a significant regulatory role in almost all differential pathways. Among the five types of RNA modifications, m5c was identified as regulating the most genes. In the Calcium signaling pathway, for instance, a total of 21 genes were found to be regulated by m5c. Importantly, we noted that Arachidonic acid metabolism was regulated by all five types of RNA modifications (Fig. 3C).

**Table 2:** Analysis of RNA epigenetic regulation network.

ID	Description	Gene Ratio	P value
hsa04061	Viral protein interaction with cytokine and cytokine receptor	20/340	3.4042E−09
hsa04657	IL-17 signaling pathway	18/340	4.3076E−08
hsa04060	Cytokine-cytokine receptor interaction	34/340	4.9586E−08
hsa04080	Neuroactive ligand-receptor interaction	35/340	2.2394E−06
hsa04614	Renin-angiotensin system	8/340	2.3016E−06
hsa04062	Chemokine signaling pathway	23/340	4.1335E−06
hsa05144	Malaria	10/340	3.0972E−05
hsa04970	Salivary secretion	13/340	9.8877E−05
hsa05150	Staphylococcus aureus infection	13/340	0.00015389
hsa04934	Cushing syndrome	17/340	0.0002255
hsa04261	Adrenergic signaling in cardiomyocytes	16/340	0.00047033
hsa04668	TNF signaling pathway	13/340	0.00071262
hsa04726	Serotonergic synapse	13/340	0.00091632
hsa04020	Calcium signaling pathway	21/340	0.00096803
hsa04072	Phospholipase D signaling pathway	15/340	0.00119402
hsa04380	Osteoclast differentiation	13/340	0.00244792
hsa04151	PI3K-Akt signaling pathway	26/340	0.00323659
hsa04972	Pancreatic secretion	11/340	0.00324561
hsa04540	Gap junction	10/340	0.00336685
hsa00590	Arachidonic acid metabolism	8/340	0.00349271
hsa04974	Protein digestion and absorption	11/340	0.0035026
hsa04976	Bile secretion	10/340	0.00365711
hsa05143	African trypanosomiasis	6/340	0.00382232
hsa04022	cGMP-PKG signaling pathway	15/340	0.00391273

### Comprehensive epigenetic regulation network construction

**Figure 4:**
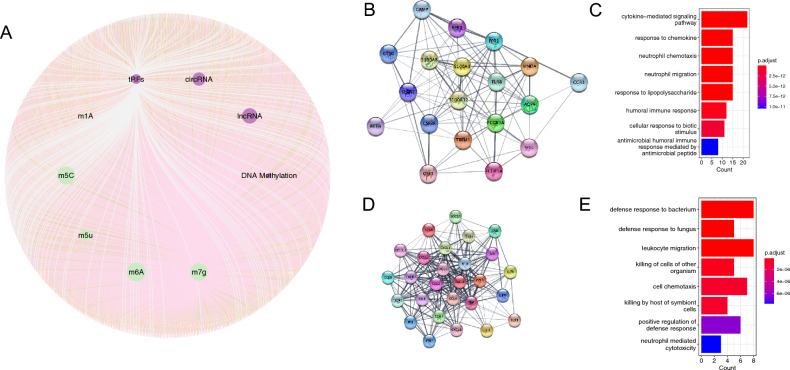
Construction of epigenetic regulation network. (**A**) Comprehensive regulation network, where the middle layer represents different epigenetic regulation modes, and the outer layer represents the genes regulated by different modes. (**B**) Construction of core module 1 network. (**C**) Functional enrichment analysis of core module 1. (**D**) Construction of core module 2 network. (**E**) Enrichment analysis of core module 2.

We established a comprehensive RNA epigenetic regulatory network by integrating two previously discussed regulatory networks. This newly constructed network encompassed 831 regulated genes, with lncRNAs regulating the most genes at the DNA epigenetic level, and m6a regulating the most genes at the RNA level (Fig. 4A). Interestingly, there were no genes that were regulated by all nine types of gene regulation, but seven genes were regulated by eight methods of regulation, including ABCB9, ALX4, BAMBI, DKK2, GREM2, MEX3B, and PMAIP1. To better understand the interactions of these regulatory genes, we performed a PPI analysis, which uncovered an average node degree of 6.91 and 2660 edges (Supplementary Fig. 2). MCODE was used to identify two core modules in the PPI network. Module 1 consisted of 27 genes and was primarily associated with cytokine response (Fig. 4B, C), while Module 2 included 19 genes and was mainly linked to cell chemotaxis (Fig. 4D, E).

### TAO core epigenetic regulatory network

**Figure 5:**
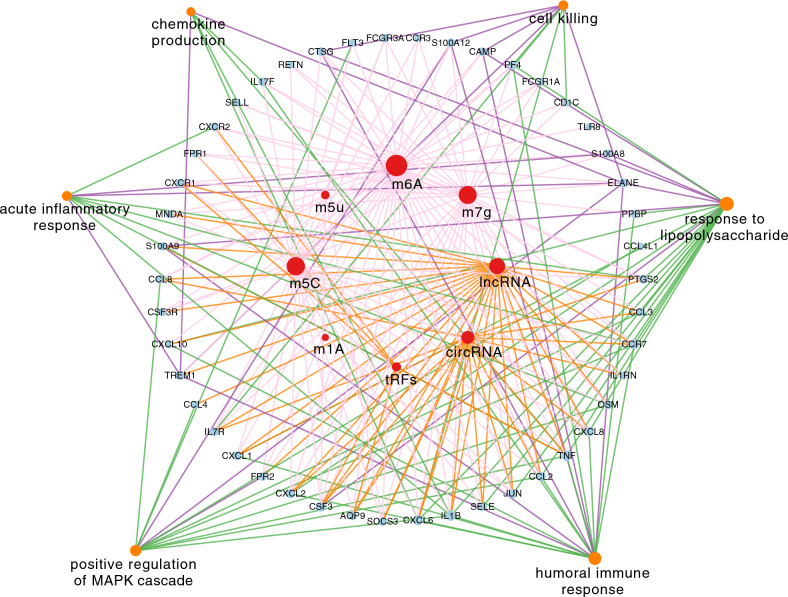
Core epigenetic regulation network of TAO. The red node in the middle represents different epigenetic regulation modes, the second layer node represents genes regulated by corresponding modes, and the outermost layer node represents the functional enrichment of regulated genes.

After a thorough analysis, we were able to identify the regulatory pathways that were shared by the two modules, and leveraged this information to construct the core epigenetic regulatory network. Through this process, we identified specific regulators for each modality, such as circRNA regulating 18, lncRNA regulating 27, m1A regulating 1, m5C regulating 34, m5u regulating 5, m6A regulating 43, m7g regulating 33, and tRFs regulating 7. The shared pathways between the two modules were related to cell killing, cytokine production, and immune response, and were all incorporated into our final network diagram (Fig. 5).

## Discussion

The pathogenesis of Thyroid-Associated Ophthalmopathy (TAO) is a complex biological process that remains incompletely elucidated, causing perplexity among numerous researchers and clinical practitioners. Currently, the diagnosis of TAO and assessment of its activity primarily rely on the patient's clinical symptoms, as there are no suitable biomarkers available for early diagnosis and differentiation of TAO. The current treatment approaches for TAO mainly involve symptomatic management, including local care, steroid pulse therapy, immunosuppressant usage, radiotherapy, and surgical interventions. However, these therapies are often accompanied by strong side effects and numerous complications. Therefore, there is an increasing interest in exploring the underlying mechanisms of TAO in order to provide biomarkers for early diagnosis, identify potential therapeutic targets for targeted blockade treatment, and establish a clearer classification of disease severity and activity, which have become significant research focuses in recent years. Furthermore, with the advancements in high-throughput sequencing technology, non-coding RNAs, especially lncRNAs and circRNAs, have been discovered to play crucial disruptive roles in transcription processes, serving as key molecules in gene regulation. Particularly, the establishment of the competitive endogenous RNA (ceRNA) hypothesis has propelled rapid developments in non-coding RNA research. However, research on epigenetics in the pathogenesis of TAO is relatively scarce. Therefore, exploring the potential roles of epigenetics in the development of TAO, attempting to construct the core epigenetic regulatory network, and searching for potential therapeutic targets and biomarkers for early diagnosis become particularly important. In our study, we investigated the specific regulatory genes of epigenetics in TAO through research on three different epigenetic regulation methods (DNA methylation, non-coding RNA, and RNA methylation). At the same time, we comprehensively screened the core regulatory genes of epigenetics by integrating the three regulatory methods.

In our current research, we took an analytical approach towards understanding the molecular mechanisms underlying Thyroid-associated ophthalmopathy (TAO). We studied the differentially expressed and differentially methylated genes in TAO and analyzed them using single omic approaches, such as DNA methylation and non-coding RNA. Through our enrichment analysis, we found that the genes in the PI3K signaling pathway were related to TAO. This pathway is critical for various cellular activities, such as cell proliferation, metabolism, and survival, by catalyzing PIP2's conversion to PIP3, which activates downstream pathways such as the Akt and mTOR signaling pathways. The PI3K-AKT signaling pathway plays a significant role in regulating cell growth and death in TAO through its involvement in proliferation and apoptosis. Recent studies have shown that orbital fibrocytes express the thyrotropin receptor (TSHR) and that activation of this receptor leads to increased expression of inflammatory genes and proliferation via the PI3K-AKT pathway. Bin Li and Terry J. Smith have demonstrated that TSH induces the production of IL-1RA in fibrocytes and GD-OFs by activating the PI3K/AKT pathway. Inhibiting either PI3K or AKT using small molecule inhibitors or by targeting their expression with small interfering RNA has been shown to attenuate the effects of TSH. ^27–29^. Moreover, inhibitors targeting this pathway have been observed to reduce inflammation and alleviate Th17 cell function, which is crucial in TAO pathogenesis, providing a new therapeutic avenue for TAO treatment^30^. A recent preclinical study has found that a PI3K signaling pathway inhibitor, called END-Uro-ONC-9, effectively inhibit the proliferation, metastasis of thyroid tumor cells, limit inflammation and promote tissue repair, making it a potential candidate for treating TAO^31^.

Subsequently, we conducted a cross-analysis between different omics to understand the expression of TAO differential genes by epigenetics at both DNA and RNA levels. Through the analysis, we found that the epigenetics at both DNA and RNA levels were involved in the regulation of the IL-17 signaling pathway. IL-17 is a multifunctional immune cytokine that is typically expressed and secreted by specific subsets of T cells (i.e., TH17 cells). IL-17 plays an important role in cell migration, inflammatory response, and immune responses, and therefore plays a crucial role in many inflammatory and autoimmune diseases^32–34^. The expression levels of IL-17 and TH17 cells are significantly increased in TAO, and the level of IL-17 is closely related to the degree of eye protrusion and inflammation^35^. The IL-17 signaling pathway plays a crucial role in numerous autoimmune and inflammatory diseases due to its ability to attract and activate various inflammatory immune cells, including neutrophils, monocytes, and lymphocytes. This results in the migration of these cells to the affected area, causing tissue damage and exacerbating the inflammatory response. IL-17A and IL-17F are key members of this pathway, binding to IL-17 receptor A and C and activating transcription factors such as NF-kB, C/EBP, AP-1, STAT3, as well as downstream signaling pathways like PI3K/AKT, ERK1/2, and p38 MAPK^36^. These signaling pathways have the potential to impact various processes, including cell growth, survival, proliferation, and inflammatory response. Previous research has shown that the combination of IL-17 and its receptors can stimulate the secretion of IL-23 by dendritic cells, leading to the differentiation of Th17 cells and the production of IL-17 in a feedback loop. The IL-23/IL-17A axis is recognized as a significant inflammatory pathway. In a study by Yuan Pan et al., it was found that PBMCs from patients with TAO secreted higher levels of IL-23 and IL-17A compared to healthy individuals, and there was a positive correlation between IL-17A and IL-23 levels. Inhibiting the secretion/expression of IL-17 has been proposed as a potential therapeutic strategy for autoimmune ocular inflammatory diseases, as demonstrated by the successful use of IL-23 and IL-17 antagonists. ^36^. A study on a bovine ocular model found that IL-17 could mediate the inflammatory response induced by TSHR autoantibodies, leading to the occurrence of TAO^37^. Recent studies have shown that by intervening in the STAT3/IL-17 signaling pathway, IL-17-induced inflammatory responses and FasL expression can be inhibited, thus alleviating the symptoms of TAO^38^]. In addition to these classical pathways, we also found that RNA epigenetics are involved in the regulation of arachidonic acid metabolism. The impact of metabolism on TAO may become a focus of our future research, in addition to its relationship with immune-related pathways.

Finally, The comprehensive impact of these epigenetic mechanisms on TAO pathogenesis is multifaceted. Epigenetic changes can influence the expression of a wide array of genes involved in immune regulation, fibroblast function, adipogenesis, and tissue remodeling. These changes can occur in response to environmental triggers, such as smoking, and can be influenced by genetic predisposition. The interplay between these epigenetic mechanisms can lead to a sustained and aberrant activation of orbital fibroblasts and immune cells, resulting in the clinical manifestations of TAO, such as proptosis, diplopia, and periorbital edema. we constructed a comprehensive epigenetic network based on DNA epigenetics and RNA epigenetics. In this regulatory network, we identified two core modules. The genes regulated by the core regulatory networks in these two modules were found to be related to immunity and inflammatory responses, which are recognized as the pathogenic mechanisms of TAO. This indirectly confirms the critical regulatory role of epigenetics in the development of TAO. With the gradual deepening of research, the impact of epigenetics on TAO is gradually increasing, and DNA methylation has been studied since the 1990s, with multiple studies demonstrating its regulatory role in TAO^39,40^ Apart from DNA methylation, research on non-coding RNA and RNA methylation is currently limited, and is a potential new direction for future research. Among the core regulatory genes, CXCL10 is regulated by multiple genes. CXCL10, also known as IP-10, is a chemokine belonging to the CXC chemokine family that can attract immune cells such as T cells, natural killer cells, and monocytes to the site of inflammation, playing an important role in the development and progression of many immune-related diseases. Recent studies have shown that CXCL10 also has a certain relationship with TAO. The expression and activity of CXCL10 have been extensively studied in TAO^41^. Some research results have shown that the expression level of CXCL10 is elevated in the serum of TAO patients and positively correlated with disease severity and progression^42^. In addition, some literature reports on the expression of CXCL10 in the eye tissues of TAO patients. CXCL10 promotes the aggregation of immune cells to the local TAO, thereby triggering the development and progression of TAO. Recently, some CXCL10 inhibitors such as teprotumumab have entered clinical applications and have achieved good therapeutic effects in the treatment of TAO^43^. Apart from CXCL10, other genes identified in the same pathway, such as CXCL1, have not been studied.

In conclusion, our analysis of various epigenetic methods using multiple omics sequencing data provided insights into the regulation of TAO. By integrating multiple epigenetic regulation methods, we identified the core genes related to TAO, which may provide innovative ideas and potential genes for research by other authors to identify TAO biomarkers and potential therapeutic targets. Our research has identified a potential new area for future studies of epigenetics in TAO. Our team and members of other laboratories should use this network and conduct more studies to further validate these findings.

### Supplementary Information

Supplementary Figures.

## Data Availability

The datasets presented in this study can be found in online repositories. The names of the repository/repositories and accession number(s) can be found below: https://www.ncbi.nlm.nih.gov/geo/query/acc.cgi?acc=GSE175399; https://www.ncbi.nlm.nih.gov/geo/query/acc.cgi?acc=GSE186480; https://www.ncbi.nlm.nih.gov/geo/query/acc.cgi?acc=GSE185952.
