# Supplementary material for: A comprehensive epigenetic network can influence the occurrence of thyroid-associated ophthalmopathy by affecting immune and inflammatory response
Source: Sci Rep. 2024 Jun 12;14:13545. doi: 10.1038/s41598-024-64415-8 (PMC11169257; doi:10.1038/s41598-024-64415-8)
Supplement: Supplementary file 1 — Supplementary Figures. [file 41598_2024_64415_MOESM1_ESM.docx]

Supplementary Figure 1: Differential expression analysis volcano plot


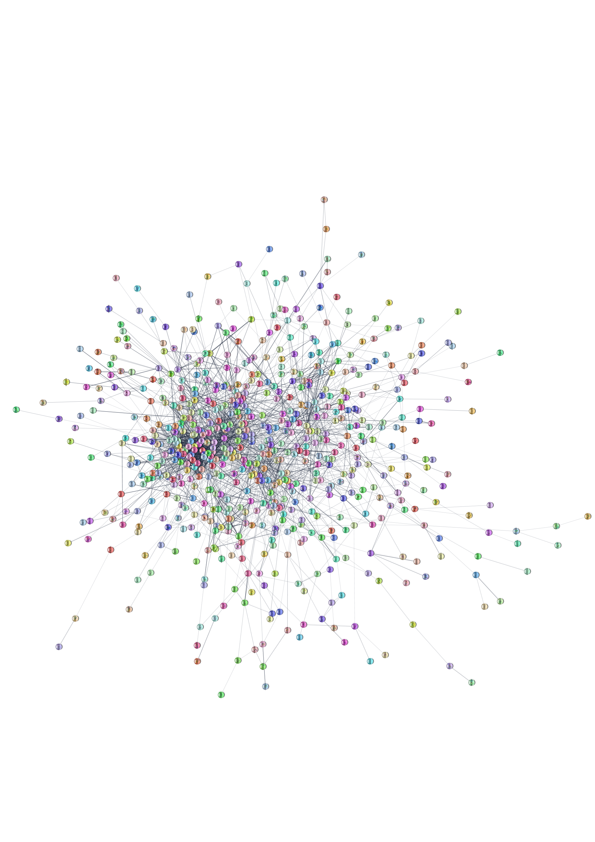


Supplementary Figure 2: Protein-Protein Interaction Networks of Epigenetic regulated genes
